# Gender-Based and Sexualized Behaviors in Perioperative Workplaces in Türkiye: An Exploratory Cross-Sectional Survey

**DOI:** 10.3390/healthcare14193271

**Published:** 2026-10-02

**Authors:** Semin Turhan, Sibel Önen Özdemir, Selçuk Kayır, Güvenç Doğan, Özgür Yağan, Volkan Hancı

**Affiliations:** 1 Department of Anesthesiology and Reanimation, Hitit University Erol Olçok Training and Research Hospital, Çepni Mahallesi, İnönü Caddesi No. 176, Çorum 19030, Türkiye; sibelonen89@gmail.com; 2 Department of Anesthesiology and Reanimation, Faculty of Medicine, Hitit University, Çorum 19030, Türkiye; drskayir@gmail.com (S.K.); guvencdogan@gmail.com (G.D.); ozguryagan@hotmail.com (Ö.Y.); 3 Department of Anesthesiology and Reanimation, Faculty of Medicine, Dokuz Eylül University, İzmir 35330, Türkiye; vhanci@gmail.com

**Keywords:** anesthesiology, gender-based harassment, healthcare personnel, operating rooms, organizational climate, sexual harassment, workplace behavior

## Abstract

**Highlights:**

**What are the main findings?**

- In this self-selected perioperative sample, 15/176 respondents (8.5%) reported 12-month personal exposure; all 15 were women.
- Nearly two thirds experienced or witnessed at least one specified behavior, while reporting was uncommon among those routed to reporting questions.

**What are the implications of the main findings?**

- Institutional responses should prioritize accountable leadership, protection against retaliation, and trusted reporting pathways.
- The findings are exploratory and require confirmation using validated instruments and denominator-known sampling.

**Abstract:**

Background/Objectives: Evidence on gender-based and sexualized conduct in perioperative workplaces in Türkiye is limited. This exploratory study described 12-month self-identified personal exposure, behavior encounters, reporting, organizational climate, impacts, and prevention priorities in a self-selected sample. Methods: An anonymous web survey used open non-probability recruitment from 8 June to 31 July 2026. Eligible respondents worked in operating-room or anesthesia services. Sample proportions were reported with Wilson 95% confidence intervals (CIs), and sex comparisons were performed. Results: Of 183 submissions, 179 were eligible (111 women; 68 men). Self-identified personal exposure to either gender-based or sexual harassment was reported by 15/176 respondents (8.5%, 95% CI 5.2–13.6): 13.8% of women and 0% of men (*p* < 0.001). Overall, 63.1% reported experiencing or witnessing at least one specified behavior; this secondary outcome was not a measure of personal harassment prevalence. Among 75 respondents routed through the context items to the reporting question, 74.7% selected ‘No’ or ‘Considered reporting but did not’; the item did not identify which specific behavior was being considered. Lower exploratory climate scores and higher impact scores accompanied personal exposure. Managerial sanctions and retaliation protection were rated the highest. Conclusions: Within this self-selected sample, the observed conduct, sex differences, and limited use of reporting pathways within the routed subgroup warrant organizational attention. Results should not be interpreted as nationally representative prevalence estimates.

## 1. Introduction

Gender-based and sexual harassment are occupational hazards with consequences for psychological well-being, professional development, workforce retention, and organizational functioning [1]. Sexual harassment is commonly conceptualized as gender harassment, unwanted sexual attention, and sexual coercion. In this study, gender-based behaviors refer to disrespect, hostility, or exclusion linked to sex or gender, whereas sexualized behaviors refer to comments, jokes, advances, messages, or physical contact with sexual content or connotation. These behavioral descriptions do not, by themselves, establish intent, severity, or a legal or institutional determination of harassment [1,2]. A meta-analysis found that 59.4% of medical trainees had experienced at least one form of harassment or discrimination, whereas a global review estimated 12-month sexual-harassment exposure among healthcare workers at 12.4%; heterogeneity across studies reflects differences in populations, settings, definitions, and measurement [3,4].

The perioperative workplace may be especially vulnerable because multidisciplinary teams work under time pressure within pronounced professional hierarchies. Disruptive behavior can impair team communication and performance, and specialty-specific evidence shows substantial gender differences. In academic anesthesiology in the United States and Canada, Hastie et al. reported 12-month sexual-harassment exposure in 43.2% of women and 14.5% of men [5]. A 2025 French anesthesia survey likewise identified widespread operating-room incivility and more verbal aggression, sexual harassment, and deviant behavior among women [6]. A multicenter study of operating-room nurses documented sexual harassment and associated occupational factors [7]. In Türkiye, sexual harassment among nurses has been described, but evidence spanning anesthesia and operating-room personnel, reporting behavior, and perceived organizational response remains limited [8].

Organizational context is central to understanding these events. Lack of trust, fear of retaliation, ambiguity about reporting, and perceived managerial inaction can suppress disclosure [9,10,11]. Conversely, psychosocial safety climate, credible procedures, support for affected workers, and consistent accountability may help prevent harm and promote reporting [12]. Existing perioperative studies have largely focused on single professions or academic anesthesia and have not jointly characterized global self-identified exposure, behaviorally anchored encounters, reporting, organizational climate, perceived impacts, and prevention priorities in a multiprofessional Turkish sample. Accordingly, our primary objective was to describe 12-month personal exposure to gender-based or sexual harassment within a self-selected sample of operating-room and anesthesia personnel and compare the observed proportions by sex. Secondary objectives were to characterize witnessing and specified behaviors; describe sources, settings, and power relationships; assess reporting, exploratory institutional-climate and impact item sets, and prevention priorities; and summarize open-ended recommendations. The study was not designed to estimate nationally representative prevalence.

## 2. Materials and Methods

### 2.1. Study Design and Reporting

This exploratory, cross-sectional, anonymous web survey was conducted in Türkiye between 8 June and 31 July 2026. The study included a quantitative questionnaire (Supplementary File S1) and one open-ended item soliciting participants’ recommendations. The protocol was not prospectively registered. Reporting followed the Strengthening the Reporting of Observational Studies in Epidemiology (STROBE) statement, the Consensus-Based Checklist for Reporting of Survey Studies (CROSS), and the Checklist for Reporting Results of Internet E-Surveys (CHERRIES) [13,14,15].

### 2.2. Participants and Eligibility

The target population comprised health professionals and other personnel actively working in operating-room and/or anesthesia services in Türkiye. Eligibility was determined by the first screening question, which asked whether the respondent was currently active in these services; respondents selecting ‘No’ were excluded. Recruitment was nationwide and was not restricted to one center. However, no directly identifying information, including names or institution names, was collected; therefore, the number of distinct contributing institutions could not be determined.

### 2.3. Recruitment and Survey Administration

The survey link was disseminated through an open, non-probability recruitment strategy. With the knowledge of the Turkish Society of Anaesthesiology and Reanimation, the invitation was emailed to society members and shared through professional WhatsApp and society-related messaging groups. The link could be redistributed. The society mailing-list size, the number of people reached through redistributed links, and survey page-view data were unavailable; consequently, the invitation denominator could not be estimated. Google Forms exported only submitted questionnaires and did not provide the number of people who opened or initiated but did not submit the survey. View, participation, completion, and response rates therefore could not be calculated. The resulting sample was self-selected and was not intended to reproduce the national distribution of perioperative professions, institutions, or regions.

The questionnaire was administered using Google Forms. Google-account sign-in and the one-response-per-account setting were used only to limit duplicate participation. The form was not configured to collect email addresses. Google-account identifiers used by the platform to enforce the single-response restriction, email addresses, and IP-related information were never visible to, accessed by, exported to, or retained by the research team. No name, institution name, or other directly identifying field was requested. No incentive was offered, and completion time was not formally recorded. Participation was voluntary, and proceeding with and submitting the questionnaire constituted electronic informed consent.

### 2.4. Questionnaire Development

All questionnaire items were developed de novo by the investigators within a literature-informed framework and were not adapted from a copyrighted scale. Content and wording were reviewed by a panel of 10 specialists: five author-anesthesiologists, two additional non-author anesthesiologists, and three non-author surgeons. The panel qualitatively assessed relevance, clarity, response-option suitability, and perioperative applicability; revisions were made by discussion and consensus. This process constituted qualitative content and face review rather than psychometric validation. No quantitative content-validity index or ratio was calculated, and construct validity, criterion validity, and test–retest reliability were not evaluated.

More specifically, the content domains were anchored to the National Academies’ framework distinguishing gender harassment, unwanted sexual attention, and sexual coercion [1]. Recent healthcare and perioperative surveys informed the 12-month timeframe and the domains covering behaviorally anchored encounters, witnessing, sources, settings, consequences, and reporting [5,7,8,11]. Evidence on reporting barriers and organizational climate informed items addressing retaliation, reporting knowledge, managerial response, support, and institutional follow-through [9,10,12], while qualitative prevention research informed the candidate intervention items [16]. The questionnaire remained study-specific: no item wording, scoring rule, or validated scale was copied or adapted from these sources.

After expert review, the questionnaire was pilot-tested with 12 health professionals representing the target population to assess comprehensibility, clarity of language, suitability of response options, and survey flow. Minor wording and formatting changes were made in response to feedback. Pilot participants were excluded from the final sample. The pilot assessed usability and did not establish validity or reliability. Accordingly, the questionnaire should be regarded as a study-specific, literature-informed item set rather than a psychometrically validated measurement instrument.

### 2.5. Questionnaire Domains

Eligibility and characteristics. Current employment in operating room/anesthesia services, geographic region, professional role, sex, age group, professional experience, institution type, primary work unit, and shift work.

Institutional climate. Five investigator-developed items scored from 1 (strongly disagree) to 5 (strongly agree). Higher values indicate a more favorable perceived institutional climate; any composite was treated as exploratory.

Primary exposure. Personal exposure to gender-based or sexual harassment during the previous 12 months (Yes/No/Prefer not to answer).

Witnessing. Witnessing sexual harassment directed at another person during the previous 12 months (Yes/No/Unsure).

Specified behaviors. Six behaviorally anchored items ranging from gender-based disrespect and exclusion to unwanted advances and physical contact, with ordinal frequency responses from never to more than once weekly.

Operational terminology. For analysis, gender-based behaviors comprised belittling or disrespect and exclusion because of sex or gender (items C3.1–C3.2). Sexualized behaviors comprised uncomfortable comments about appearance or private life, sexual jokes or insinuations, unwanted flirting, messages or offers, and unwanted physical contact (items C3.3–C3.6). Verbal behaviors encompassed comments, jokes, and insinuations; ‘unwanted’ reflected the respondent’s appraisal. These behavior items represented reported encounters and were not assumed to meet a legal or institutional threshold for harassment.

Context and consequences. Source, setting, power relationship, six investigator-developed perceived-impact items, reporting behavior, and reasons for non-reporting. Any impact composite was treated as exploratory.

Prevention. Perceived effectiveness of five preventive strategies and one technically mandatory open-ended recommendation item. The open-ended field could be completed with a non-substantive entry if a participant did not wish to provide a recommendation.

### 2.6. Sample Size

The sample size calculation was based on the 12-month sex-specific exposure proportions reported by Hastie et al. (43.2% among women and 14.5% among men) [5]. These proportions correspond to a Cohen h of 0.653. With a two-sided alpha of 0.05, 80% power, and equal allocation, the minimum required sample was 37 participants per sex group (74 in total); a 90% power scenario required approximately 50 per group (100 in total). The study by Hastie et al. provided the closest available sex-stratified perioperative estimate and was used only as an external planning benchmark for a group contrast, not as an expected prevalence for Türkiye. Differences in population, profession, setting, and outcome definition make the assumed effect size uncertain. The final dataset included 179 eligible respondents; 176 provided an analyzable primary-outcome response, including 109 women and 67 men. This calculation did not establish precision or representativeness for national prevalence estimation; observed estimates are therefore reported with confidence intervals and interpreted within the analyzed sample.

### 2.7. Ethical Considerations

The study was approved by the Hitit University Faculty of Medicine Research Ethics Committee at its meeting on 31 March 2026 (decision no. 2026-46). Participation was voluntary and anonymous. Before beginning the questionnaire, participants viewed an information page explaining the study purpose, the sensitive nature of the questions, anonymity, the right to discontinue before submission, and the use of data solely for aggregate scientific analysis. The survey did not permit every item to be left blank. In particular, the final open-ended recommendation field was technically mandatory, but respondents could enter a non-substantive response such as ‘none’ or punctuation if they did not wish to provide a recommendation. Voluntarily proceeding with and submitting the anonymous questionnaire constituted electronic informed consent. Because no identifying link was retained, an individual response could not be located and withdrawn after submission.

The de-identified data were stored for five years in a password-protected, access-restricted digital archive accessible only to the research team. No dedicated psychological-support or institutional-referral contact was embedded in the survey; this is acknowledged as a limitation for research on a sensitive topic.

### 2.8. Outcome Definitions

Primary outcome. A ‘Yes’ response to the single item asking about personal exposure to gender-based or sexual harassment during the previous 12 months. ‘Prefer not to answer’ responses were excluded from the denominator. The item combined the two constructs and relied on respondents’ own identification of an event as harassment; it did not determine whether the event was gender-based harassment, sexual harassment, or both, and it did not independently adjudicate a legal or institutional threshold. The resulting percentage was therefore interpreted as an observed within-sample proportion of self-identified exposure to either category, not as construct-specific or population prevalence. This variable and its comparison by sex constituted the primary outcome and primary analysis; witnessing, specified behaviors, context, reporting, climate, impact, prevention, and qualitative themes were secondary or exploratory analyses.

Witnessing: A ‘Yes’ response defined definite witnessing. A sensitivity analysis combined ‘Yes’ and ‘Unsure’ responses.

Specified behavior encounter: Any response other than ‘Never’ to at least one of the six behavior items. Unlike the primary outcome, this variable did not require respondents to label an event as harassment and it combined personal experience with witnessing. It was therefore treated as a broader behavior-encounter measure and was not interpreted as personal harassment prevalence or as an alternative estimate of the primary outcome.

Frequent specified behavior: At least one specified behavior reported more than once per month or more than once per week.

Reporting: The reporting questions were not triggered by a positive response to any single primary, witnessing, or specified-behavior item. In the context section, respondents selecting ‘I did not experience such a situation’ at the setting or power-relationship routing items were sent directly to the prevention section (Question 29); respondents giving a substantive context response proceeded through the effects and reporting sections. The reporting denominator comprised the 75 respondents who reached and completed the reporting item. ‘No’ and ‘Considered reporting but did not’ were combined for the principal descriptive summary. Because the item referred globally to ‘the situation that you experienced or witnessed’ and was not linked to a particular behavior, the resulting percentage does not establish that every endorsed behavior met a formal reporting threshold or identify which event a respondent considered reporting.

### 2.9. Data Management

The raw data files were preserved unchanged. Google Forms exported only submitted questionnaires; abandoned or unsubmitted starts were not available. The exported files contained no name, institution name, email address, IP-related information, or Google-account identifier. Of 183 submitted records, four respondents selected ‘No’ on the occupational eligibility screen and were excluded. No submitted questionnaire was excluded because of incompleteness or invalidity, leaving 179 eligible participants in the final analytic sample. Questionnaire routing produced expected structural missingness, which was not treated as an incomplete submission and was not imputed. Three participants selected ‘Prefer not to answer’ for the primary outcome, producing a primary-outcome denominator of 176. Quantitative variables were retained from the original 39-column export. The technically mandatory open-ended item was merged from the second export by timestamp; respondents could submit a non-substantive entry, and this preserved two regional values present in the original export but absent from the later file. The Google Forms single-response setting was used to reduce duplicate participation; no record was removed solely because its categorical response pattern matched another record.

### 2.10. Statistical Analysis

Categorical variables were summarized as counts and percentages. Observed proportions were reported with 95% Wilson confidence intervals (CIs). These CIs quantify uncertainty around analyzed responses but do not account for selection arising from non-probability recruitment. Ordinal Likert-type items were described using response distributions and medians with interquartile ranges; means and standard deviations were also supplied for comparison when clearly labeled. Cronbach alpha was used only to summarize within-sample internal consistency of the investigator-developed institutional-climate and perceived-impact item sets; it was not interpreted as evidence of construct validity. Composite-score analyses were exploratory. Benjamini–Hochberg false-discovery-rate correction was applied separately to two prespecified families of secondary comparisons: the six individual behavior-level sex comparisons and the five prevention-strategy sex comparisons. Unadjusted *p* values and adjusted q values are reported in Tables, respectively; multiplicity correction was not applied across all secondary analyses.

The primary comparison evaluated personal exposure by sex using Fisher’s exact test. Because no man reported the primary outcome and only 15 primary events occurred, no multivariable model was fitted for personal exposure. Firth penalized logistic regression was considered as a possible sparse-data alternative, but an adjusted model was not pursued because the limited event count and complete separation by sex would still yield imprecise, assumption-sensitive inference. A multivariable model was also not fitted for the broader specified-behavior outcome because it combined personal experience and witnessing and therefore did not support construct-specific interpretation of adjusted odds ratios. Sex-stratified observed proportions and exact comparisons were emphasized. Secondary categorical comparisons used Fisher’s exact or chi-square tests as appropriate. Ordinal and composite scores were compared with the Mann–Whitney U test. A reviewer-requested post hoc analysis compared the exploratory institutional-climate composite between respondents who reported and those who did not report or considered but did not report; reason-specific comparisons were not performed because several reason categories contained very few respondents.

Tests were two-sided, with *p* < 0.05. Missing values were not imputed. Analyses were performed in Python 3.12.13 using pandas 2.2.3, NumPy 2.3.5, and SciPy 1.17.0.

### 2.11. Open-Ended Recommendation Analysis

Open-ended recommendations underwent exploratory hybrid deductive–inductive content analysis. Semin Turhan and Selçuk Kayır independently reviewed only the de-identified recommendation texts and were masked to participants’ demographic characteristics and quantitative responses. A literature-informed initial framework was applied, and new themes were added when warranted by the data. The open-ended field was technically mandatory, but respondents could enter a non-substantive response rather than provide a recommendation. Entries without an interpretable recommendation (e.g., punctuation, ‘none’, or a statement of no exposure without a recommendation) were classified as non-substantive. Substantive responses could receive more than one code. Coding disagreements were resolved by discussion and consensus. The final framework comprised accountability and sanctions; education and awareness; safe and accessible reporting; leadership, impartiality, and hierarchy; respectful and equitable culture; victim protection and support; and monitoring, security, and process controls.

### 2.12. AI-Assisted Manuscript Editing

OpenAI ChatGPT/Codex (OpenAI, GPT-5.6 Sol version, San Francisco, CA, USA; accessed 21 September 2026) was utilized during the process of refining the English language. These tools were not used in the stages of study design, data collection, qualitative coding, source selection, or autonomous scientific interpretation. The authors, who determined the analyses and independently reviewed the codes, numerical outputs, edited text, and interpretations, bear full responsibility for the study.

## 3. Results

### 3.1. Participant Flow and Characteristics

A total of 183 questionnaires were submitted (Figure 1). The platform export did not contain abandoned survey starts, and the invitation denominator and page-view count were unavailable. Four respondents did not meet the occupational eligibility criterion and were excluded; no submitted form was excluded for incompleteness or invalidity. The final self-selected analytic sample therefore comprised 179 participants, including 111 women (62.0%) and 68 men (38.0%). Anesthesiologists (65/179, 36.3%) and academic anesthesiologists/faculty members (36/179, 20.1%) were the largest professional categories; all original professional-role and professional-experience categories are presented in Table 1. All seven geographic regions were represented, with the largest contributions from Central Anatolia (31.3%) and the Aegean region (20.7%). Participants reported employment in university hospitals (55/179, 30.7%), training and research hospitals (80/179, 44.7%), state hospitals (33/179, 18.4%), and private hospitals (11/179, 6.1%). Institution names were not collected, so the number of distinct participating centers could not be established. Most respondents worked primarily in the operating room (163/179, 91.1%), and 138 (77.1%) undertook shift or on-call work.

Participant characteristics are summarized in Table 1.

### 3.2. Primary and Secondary Outcomes

Three participants selected ‘Prefer not to answer’ for the primary outcome. Among 176 analyzable responses, 15 reported personal exposure to gender-based or sexual harassment during the previous 12 months (observed proportion 8.5%, 95% CI 5.2–13.6%). All 15 reports were from women. The observed proportion was 13.8% among women (15/109; 95% CI 8.5–21.5%) and 0% among men (0/67; 95% CI 0–5.4%), corresponding to an absolute difference of 13.8 percentage points (95% CI 6.2–21.5; Fisher’s exact *p* < 0.001). Given the 15 events and zero-event male subgroup, no adjusted model was fitted for this outcome; the observed zero among men should not be interpreted as evidence that exposure does not occur among men.

Definite witnessing was reported by 33 respondents (18.4%, 95% CI 13.4–24.8%), without a statistically significant difference between women and men (21.6% vs. 13.2%; *p* = 0.172). When ‘Unsure’ responses were combined with definite witnessing, the observed proportion was 30.2% (54/179; 95% CI 23.9–37.3%). Overall, 113 participants (63.1%, 95% CI 55.9–69.9%) reported experiencing or witnessing at least one specified behavior, and 49 (27.4%, 95% CI 21.4–34.3%) reported at least one behavior monthly or more frequently. Any specified behavior was more common among women than men (69.4% vs. 52.9%; *p* = 0.038). The 8.5% primary-outcome proportion and the 63.1% specified-behavior proportion are therefore not competing estimates of the same construct: the former required self-identification of personal harassment, whereas the latter captured any personal experience or witnessing of the six listed behaviors.

Primary and secondary outcome estimates are summarized in Table 2.

### 3.3. Specified Behaviors

The most frequently reported individual behavior was sexual jokes or insinuations (77/179, 43.0%), followed by uncomfortable comments about appearance or private life (72/179, 40.2%) and gender-based disrespect (65/179, 36.3%). After Benjamini–Hochberg correction across the six behavior comparisons, gender-based disrespect (54.1% among women vs. 7.4% among men; q < 0.001) and gender-based exclusion (45.9% vs. 11.8%; q < 0.001) remained associated with sex. The other four behavior-level comparisons were not statistically significant after correction.

Behavior-specific estimates are presented in Table 3.

### 3.4. Context, Consequences, and Reporting

Among respondents who identified a setting, the operating room was the most common location (66/85, 77.6%). Among 83 respondents who selected at least one source, surgical physicians were the most frequently identified source (50/83, 60.2%); multiple responses were permitted. Of 75 respondents who completed the power-relationship item, 39 (52.0%) reported that the source occupied a superior position. These 75 respondents also completed the global reporting item: 19 (25.3%) selected ‘Yes’, and 56 (74.7%) selected ‘No’ or ‘I considered reporting but did not’. Among 55 of the latter respondents who supplied a reason, the most common was the belief that nothing would change (35/55, 63.6%). Because the reporting item was not linked to a specific behavior or event, the 74.7% describes response choices in this routed subgroup; it should not be interpreted as the proportion of all behavior endorsements, or of all formally reportable harassment events, that went unreported.

Within-sample Cronbach alpha was 0.772 for the five institutional-climate items. The exploratory composite climate score was lower among women than men (mean 2.66 vs. 3.11; Mann–Whitney U = 2908.5, *p* = 0.010) and among respondents reporting personal exposure than those denying exposure (mean 1.83 vs. 2.93; U = 466.5, *p* < 0.001). In the reviewer-requested post hoc analysis of 75 respondents routed to reporting questions, the climate composite did not differ between reporters (n = 19; median 2.2, IQR 1.5–3.4) and non-reporters or those who considered but did not report (n = 56; median 2.6, IQR 1.8–3.2; U = 493.5, *p* = 0.643). Within-sample alpha was 0.917 for the six perceived-impact items. Among the 75 routed respondents, the mean exploratory impact score was 3.31 (SD 1.22); reduced trust in the institution or team received the highest item rating (mean 3.79, SD 1.39). Impact scores were higher among women than men (mean 3.53 vs. 2.51; *p* = 0.005) and among respondents reporting personal exposure than those denying it (mean 4.13 vs. 3.06; *p* = 0.002). These analyses describe within-sample associations and do not validate the item sets.

Managerial sanctions were rated as effective or very effective by 131 participants (73.2%), followed by protection against retaliation (117/179, 65.4%), psychological support (99/179, 55.3%), and anonymous reporting (96/179, 53.6%). Mandatory education had the lowest top-two rating (60/179, 33.5%). None of the sex comparisons across the five prevention strategies remained statistically significant after false-discovery-rate adjustment. The corresponding unadjusted *p* values and Benjamini–Hochberg-adjusted q values are reported in Supplementary Table S1.

Contextual, reporting, climate, impact, and prevention findings are summarized in Table 4.

### 3.5. Open-Ended Recommendations

All 179 eligible respondents entered text in the technically mandatory open-ended field. The field could not be left blank, but respondents could continue by entering a non-substantive response if they did not wish to provide a recommendation. Fifty-four entries, including ‘none’, punctuation, or statements without an interpretable recommendation, were classified as non-substantive, leaving 125 responses containing an interpretable recommendation. Multiple themes could be assigned to each response. Accountability and sanctions was the dominant theme (56/125, 44.8%), followed by education and awareness (33/125, 26.4%); safe and accessible reporting (27/125, 21.6%); leadership, impartiality, and hierarchy (25/125, 20.0%); respectful and equitable culture (17/125, 13.6%); victim protection and support (15/125, 12.0%); and monitoring, security, and process controls (9/125, 7.2%). Percentages exceed 100% because multiple themes could be assigned.

## 4. Discussion

### 4.1. Principal Findings

In this self-selected sample of 179 operating-room and anesthesia personnel, 8.5% of respondents with analyzable primary-outcome data reported personal exposure to gender-based or sexual harassment in the previous 12 months. All 15 reports came from women, corresponding to an observed proportion of 13.8% among women and an absolute sex difference of 13.8 percentage points. Because only 15 events occurred and no event was reported by men, this pattern should be treated as a sparse within-sample observation rather than evidence that men experience no such conduct. Non-disclosure, differing interpretations, and the composition of the self-selected sample may have contributed to the observed zero. Behaviorally anchored items identified a broader pattern: 63.1% had experienced or witnessed at least one specified behavior, and more than one quarter reported a behavior monthly or more often. Gender-based disrespect and exclusion showed the clearest sex differences after multiplicity correction. The study’s contribution lies in jointly describing self-identified personal exposure, behavior encounters, power relationships, reporting, exploratory climate and impact measures, and prevention priorities within a multiprofessional perioperative sample, not in estimating national prevalence. Accordingly, the numerical gap between 8.5% and 63.1% reflects different question formats and construct breadth and should not be interpreted as disagreement between two estimates of personal harassment.

### 4.2. Comparison with Previous Research

The observed 12-month personal-exposure proportion among women was lower than the 43.2% reported by Hastie et al. in academic anesthesiology [5]. Direct numerical comparison is inappropriate because the source study sampled academic anesthesiologists in the United States and Canada, whereas our sample spanned professions and institution types and used a combined gender-based or sexual-harassment item. Participation pathways, occupational composition, social context, and respondents’ thresholds for labeling behavior also differed. The French anesthesia survey reported incivility in 83% of respondents and greater sexual harassment and verbal aggression among women, supporting the relevance of gendered exposure in perioperative environments despite different instruments and outcomes [6].

Two recent perioperative studies further illustrate why absolute rates should be compared cautiously. In a 2024 multicenter convenience sample of operating-room nurses in China, Tang et al. reported 12-month sexual harassment in 63.0%, predominantly gender harassment (56.7%), and 47.8% of affected nurses remained silent [7]. Their nurse-only population, validated instrument, region-specific recruitment, and behavior-based case definition differ materially from our combined multiprofessional exposure item. A 2026 Turkish online study of 302 surgical nurses reported prior sexual violence in 48.1%; patients and relatives were the most commonly identified perpetrators (59.3%), and 40.7% reported harassment to a manager [17]. That study focused on patient- or client-initiated behavior and did not use our 12-month multiprofessional case definition, which may help explain its different rate and perpetrator profile.

The broader occupational-health literature identifies female healthcare workers as a group at increased risk of workplace violence and harassment [18]. Operating-room nurses have reported sexual harassment across multiple institutions, and earlier Turkish nursing research found that physicians were a prominent reported source [7,8]. These studies differ in population, definitions, and measurement, but they support examining harassment as both a gendered and organizational workplace hazard.

Recent population-based Swedish data provide a contrasting sampling frame. In a weighted survey of nurses and physicians, past-year gender-based harassment was reported by 30.1% of female physicians and 8.3% of male physicians, and exposure patterns varied by profession and perpetrator source [19]. Although not perioperative, those findings support the interpretation that sex and professional position intersect while also showing why estimates derived from probability sampling and construct-specific items should not be pooled directly with estimates from the present self-selected survey.

The behavior-level pattern is consistent with evidence that gender harassment, including hostility, disrespect, exclusion, and objectification, is more common than coercive or overtly sexual conduct [1,2]. Sexual jokes were the most frequently endorsed individual behavior overall, but the strongest sex differences concerned disrespect and exclusion. Similar findings in academic medicine have linked gender-based hostility with poorer mental health, job satisfaction, safety, and turnover intentions [11,12,20]. Because several professional-role categories were small and the broader behavior outcome combined personal experience with witnessing, we did not estimate adjusted profession-specific associations.

### 4.3. Hierarchy, Reporting, and Organizational Climate

Among respondents completing the relevant routed items, the operating room was the most common setting, surgical physicians were the most frequently identified source, and a superior power position was reported by 52.0%. These within-sample findings locate respondents’ concerns within interprofessional and hierarchical relations rather than a single occupational group. Hierarchy can normalize harmful conduct and make speaking up costly, particularly when the alleged source influences training, scheduling, evaluation, promotion, or access to opportunities [10]. Among the 75 respondents routed to the reporting item, three quarters selected ‘No’ or indicated that they considered reporting but did not proceed. The dominant stated reason was the belief that nothing would change, which is descriptively relevant to institutional confidence and perceived follow-through. However, the reporting item referred globally to ‘the situation’ and was not linked to a specific endorsed behavior, its severity, or whether the respondent had personally experienced or witnessed it. Hearing a joke, witnessing conduct, and experiencing unwanted physical contact may carry different reporting expectations. The 74.7% figure therefore should not be read as evidence that all endorsed behaviors required formal reporting or that 74.7% of formally reportable harassment events went unreported. Fear of retaliation, uncertainty about what qualifies, and lack of trust in reporting systems remain recurring barriers in medical workplaces [9].

Exploratory institutional-climate scores were lower among women and among those reporting personal exposure; impact scores were higher among women and exposed respondents. The post hoc comparison between reporters and non-reporters did not show a climate-score difference, but the small reporter subgroup and sparse reason categories limited more detailed inference. The reported power imbalance, the predominance of ‘nothing would change’ as the reason for non-reporting, and the low overall climate scores remain descriptively consistent with concerns about institutional trust. Because the study was cross-sectional, temporal or causal relationships among climate perceptions, exposure, power dynamics, and reporting cannot be established. The item sets were not validated, so score differences require cautious interpretation. Reduced trust in the institution or team was the highest-rated impact item, reinforcing the importance of visible and procedurally fair responses.

### 4.4. Implications for Practice and Policy

Participants prioritized managerial sanctions and protection against retaliation, whereas mandatory education received the lowest effectiveness rating. These preferences identify candidate organizational targets rather than tested interventions. Education may help define unacceptable conduct and reporting routes, but stand-alone compliance training is unlikely to be sufficient. Potential components for formal evaluation include confidential and accessible reporting, trained and impartial investigators, support for affected workers, proportionate consequences, protection of reporters and witnesses, and transparent aggregate feedback [16]. Perioperative leaders should also consider monitoring gender-based disrespect and exclusion alongside overt sexual behavior and ensuring that reporting routes do not depend solely on the local hierarchy.

Open-ended recommendations emphasized accountability, education, safe reporting, and impartial leadership, providing descriptive triangulation with the closed-ended priorities. This convergence does not remove selection, priming, or common-method bias. Future studies should use probability-based or denominator-known sampling where feasible, apply validated multidimensional measures, include gender-diverse response options, and evaluate whether organizational interventions change exposure, reporting confidence, worker well-being, and patient-safety outcomes over time.

### 4.5. Strengths and Limitations

Strengths include responses from all seven geographic regions; inclusion of multiple perioperative professions and institution types; behaviorally anchored items; outcome-specific denominators; confidence intervals; multiplicity adjustment; and explicit separation of personal exposure from combined experience or witnessing. The questionnaire underwent literature-informed development, multidisciplinary qualitative review, and usability pilot testing. These steps improved contextual relevance and comprehensibility but should not be interpreted as psychometric validation. The one-response-per-account setting reduced duplicate submissions. The Google-account identifiers used by the platform, email addresses, and IP-related information were never visible to, accessed by, exported to, or retained by the research team.

Several limitations are important. First, open non-probability recruitment, an unknown invitation denominator, unavailable counts of survey views and abandoned starts, self-selection, and a sample dominated by physician roles limited profession-level and population-level inference. The underrepresentation of non-physician personnel may have influenced descriptions of workplace hierarchy, exposure opportunities, sources, and reporting behavior. Although respondents came from all seven regions and several hospital types, institution names were not collected, the number of contributing centers was unknown, and the sample cannot represent all healthcare workers, perioperative units, hospital sectors, or regions in Türkiye. People with relevant experiences may have been more likely to participate, whereas fear or discomfort may have deterred others; the direction of selection bias is unknown. Second, all items were investigator-developed without formal psychometric validation. The participant information page defined the topic broadly and included advocacy-oriented language and a quotation about silence, which may have primed responses. The same behavior may also be labeled differently across respondents. Third, self-report is vulnerable to recall, social-desirability, and interpretation differences. Fourth, the primary item combined gender-based and sexual harassment, whereas the behavior items combined experience and witnessing; neither can provide construct-specific personal prevalence. The global reporting item was not linked to an exact behavior, event severity, or personal-experience versus witnessing status, so the reporting percentage cannot identify the proportion of formally reportable events that were not reported. Fifth, only 15 primary events and complete separation by sex prevented adjusted modeling of personal exposure. We did not fit a multivariable model for the broader specified-behavior outcome because it combined personal experience and witnessing, and several original professional-role categories were too small for reliable adjusted profession-specific estimation. Sixth, the external effect size used for sample-size planning came from a different population and instrument and may not reflect the true sex contrast in this sample. Seventh, only the response options ‘Woman’ and ‘Man’ were offered, limiting inclusivity. Eighth, no dedicated support or referral contact was embedded in this sensitive survey. Finally, the open-text item was technically mandatory, although respondents could submit a non-substantive entry; pre-consensus coding matrices were not retained, so inter-coder agreement could not be quantified. Future studies should use validated multidimensional instruments and denominator-known, multicenter, stratified probability sampling where feasible.

## 5. Conclusions

Within this self-selected sample of perioperative personnel, self-identified personal exposure was reported only by women, and many respondents reported experiencing or witnessing at least one specified behavior. Perceived hierarchical sources, limited use of reporting pathways within the routed subgroup, low confidence that reporting would produce action, and unfavorable climate perceptions identify candidate organizational targets: trusted reporting pathways, protection against retaliation, transparent follow-through, and leadership accountability. The findings are exploratory and should not be interpreted as nationally representative or construct-specific prevalence estimates; confirmation with validated instruments and denominator-known sampling is required.

## Figures and Tables

**Figure 1:**
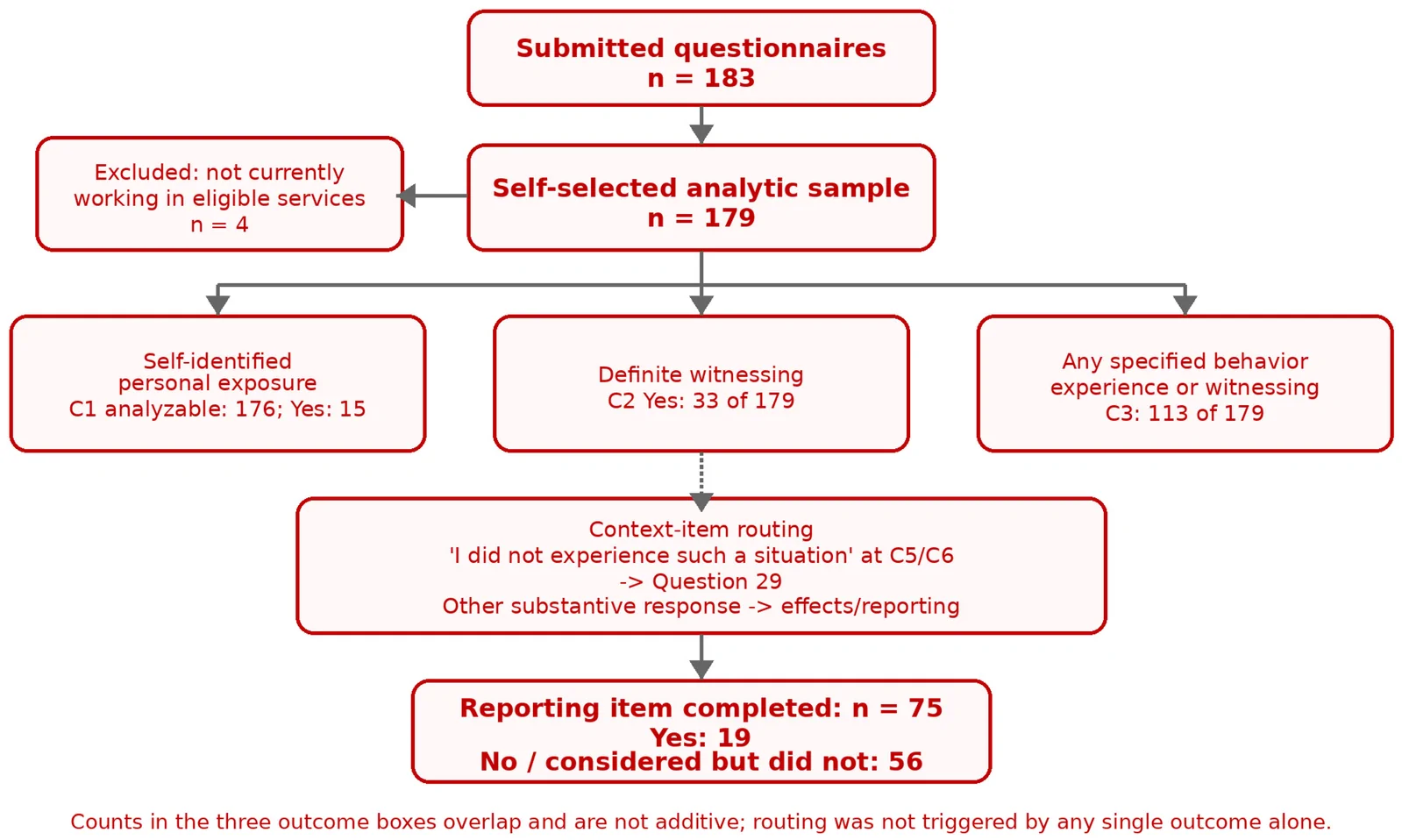
Participant, outcome, and reporting-question flow. Outcome counts overlap and are not additive. Reporting-section eligibility was determined by context-item routing rather than by any single outcome response.

**Table 1:** Participant characteristics of the self-selected analytic sample (N = 179).

Characteristic	Category	n (%)
Sex	Women	111 (62.0)
	Men	68 (38.0)
Age group	20–29 years	21 (11.7)
	30–39 years	79 (44.1)
	40–49 years	45 (25.1)
	50 years or older	34 (19.0)
Professional experience	0–2 years	10 (5.6)
	3–5 years	20 (11.2)
	6–10 years	55 (30.7)
	11–15 years	23 (12.8)
	16 years or more	71 (39.7)
Professional role (reported)	Anesthesiologist	65 (36.3)
	Academic anesthesiologist/faculty member	36 (20.1)
	Anesthesia technician/technologist	26 (14.5)
	Anesthesiology resident	21 (11.7)
	Operating-room nurse	16 (8.9)
	Intensive-care nurse/worker	4 (2.2)
	Surgeon	2 (1.1)
	Recovery nurse	2 (1.1)
	Non-health-care OR worker	2 (1.1)
	Technician	1 (0.6)
	Physician	1 (0.6)
	Resident	1 (0.6)
	General surgery resident	1 (0.6)
	General surgeon	1 (0.6)
Institution	University hospital	55 (30.7)
	Training and research hospital	80 (44.7)
	State hospital	33 (18.4)
	Private hospital	11 (6.1)
Primary unit	Operating room	163 (91.1)
Shift/on-call work	Yes	138 (77.1)

Professional experience is shown in the five response categories used in the questionnaire. Professional-role categories reproduce the prespecified options and each distinct free-text role as reported. Sparse professional categories are presented descriptively; no adjusted or profession-specific inferential model was fitted.

**Table 2:** Self-reported personal exposure and secondary outcomes stratified by sex.

Outcome	Overall, n/N (%)	Women, n/N (%)	Men, n/N (%)	*p* Value
Personal exposure, previous 12 months	15/176 (8.5)	15/109 (13.8)	0/67 (0.0)	<0.001
Definite witnessing	33/179 (18.4)	24/111 (21.6)	9/68 (13.2)	0.172
Witnessing: yes or unsure	54/179 (30.2)	37/111 (33.3)	17/68 (25.0)	0.314
Any specified behavior	113/179 (63.1)	77/111 (69.4)	36/68 (52.9)	0.038
Any behavior monthly or more	49/179 (27.4)	36/111 (32.4)	13/68 (19.1)	0.059
Gender-based disrespect/exclusion	71/179 (39.7)	63/111 (56.8)	8/68 (11.8)	<0.001
Appearance/private-life comments or sexual jokes	99/179 (55.3)	67/111 (60.4)	32/68 (47.1)	0.090
Unwanted advances/messages or physical contact	52/179 (29.1)	36/111 (32.4)	16/68 (23.5)	0.237

*p* values are from two-sided Fisher’s exact tests. Specified-behavior outcomes combine personal experience and witnessing and should not be interpreted as personal harassment prevalence.

**Table 3:** Individual specified behaviors and sex comparisons.

Behavior	Overall n (%)	Women n (%)	Men n (%)	*p* Value	FDR q Value
Gender-based disrespect	65 (36.3)	60 (54.1)	5 (7.4)	<0.001	<0.001
Gender-based exclusion	59 (33.0)	51 (45.9)	8 (11.8)	<0.001	<0.001
Appearance/private-life comments	72 (40.2)	51 (45.9)	21 (30.9)	0.059	0.119
Sexual jokes or insinuations	77 (43.0)	51 (45.9)	26 (38.2)	0.352	0.423
Unwanted flirting/messages/offers	38 (21.2)	27 (24.3)	11 (16.2)	0.259	0.388
Unwanted physical contact	41 (22.9)	27 (24.3)	14 (20.6)	0.589	0.589

*p* values are unadjusted two-sided Fisher’s exact tests. FDR q values were calculated with the Benjamini–Hochberg procedure across the six individual behavior-level sex comparisons shown in this table.

**Table 4:** Context, reporting, exploratory climate and impact scores, and prevention priorities.

Finding	n/N	Estimate
Surgical physician identified as a source	50/83	60.2%
Operating room as most frequent setting	66/85	77.6%
Source in a superior position	39/75	52.0%
Reporting item: Yes	19/75	25.3%
Reporting item: No/considered but did not	56/75	74.7%
Belief that nothing would change	35/55	63.6%
Institutional-climate composite (exploratory)	179	Mean 2.83 (SD 1.10)
Perceived-impact composite (exploratory)	75	Mean 3.31 (SD 1.22)
Managerial sanctions: effective/very effective	131/179	73.2%
Protection against retaliation: effective/very effective	117/179	65.4%

## Data Availability

The de-identified dataset and analytic code are available from the corresponding author upon reasonable scientific request, subject to ethics, privacy, and institutional requirements.

## Supplementary Materials

The following supporting information can be downloaded at: https://www.mdpi.com/article/10.3390/healthcare14193271/s1: Supplementary File S1, English translation of the investigator-developed questionnaire administered in Turkish; Supplementary Table S1, Sex comparisons for perceived effectiveness of prevention strategies.
