# Supplementary material for: Gender-Based and Sexualized Behaviors in Perioperative Workplaces in Türkiye: An Exploratory Cross-Sectional Survey
Source: Healthcare (Basel). 2026 Oct 2;14(19):3271. doi: 10.3390/healthcare14193271 (PMC13664897; doi:10.3390/healthcare14193271)
Supplement: Supplementary file 1 [file healthcare-14-03271-s001.zip › healthcare-4568501-supplementary.pdf]

Gender-Based and Sexual Harassment in the Operating Room and Anesthesia Ecosystem Survey

English translation of the Turkish questionnaire administered in the study

Translation note. This English version is provided for publication transparency and was not administered to participants. The survey was administered in Turkish. The English translation was verified by Sibel Önen Özdemir and Semin Turhan.

Development note. The questionnaire was written de novo for this study and was not copied from or scored as a published scale. The source-to-domain mapping for its literature-informed content framework is now reported in Section 2.4 of the revised manuscript.

Participant information

Dear participant,

This survey was prepared to assess health workers' experiences and observations concerning gender-based harassment, sexual harassment, inappropriate conduct, verbal or physical boundary violations, and perceptions of safety in operating-room and anesthesia workplaces.

When answering, please consider not only harassment in its commonly recognized sense, but all adverse conduct directed at a person because of sex or gender.

Participation is entirely voluntary. The study was approved by the Hitit University Faculty of Medicine Research Ethics Committee at its meeting on 31 March 2026 (decision no. 2026-46).

No name, institution name, email address, or other directly identifying information was requested. Google-account sign-in was used only to enforce the one-response-per-account restriction. Google-account identifiers, email addresses, and IP-related information were never visible to, accessed by, exported to, or retained by the research team. Data were evaluated only for scientific purposes in aggregate and anonymous form. Individual answers were not shared with third parties, institutional managers, or colleagues.

Some questions are sensitive. Participation was voluntary, and participants could stop completing the questionnaire at any time before submission. The survey did not permit every item to be left blank. In particular, the final open-ended recommendation field was technically mandatory, but a non-substantive response such as 'none' or punctuation could be entered if a participant did not wish to provide a recommendation. Starting and submitting the survey indicated voluntary participation and consent for the anonymous scientific evaluation of responses.

Your contribution will provide important information for developing a safer, more equitable, respectful, and professional culture in operating-room and anesthesia workplaces.

“Silence is the thickest wall protecting harassment.”

A. Eligibility and participant characteristics

1. A1. Are you currently working in operating-room and/or anesthesia services?

Response options: Yes; No

2. Region. In which region of Türkiye do you work?

Response options: Aegean; Mediterranean; Southeastern Anatolia; Eastern Anatolia; Black Sea; Marmara; Central Anatolia

3. A2. What is your professional role?

Response options: Operating-room nurse; Anesthesia technician/technologist; Anesthesiology resident; Anesthesiologist; Academic anesthesiologist/faculty member; Intensive-care nurse/worker; Non-health-care operating-room worker; Other

4. A3. What is your sex?

Response options: Woman; Man

5. A4. What is your age group?

Response options: 20–29; 30–39; 40–49; 50 years or older

6. A5. What is your total professional experience?

Response options: 0–2 years; 3–5 years; 6–10 years; 11–15 years; 16 years or more

7. A6. What type of institution do you work in?

Response options: University hospital; Training and research hospital; State hospital; Private hospital

8. A7. In which unit do you work most frequently?

Response options: Operating room; Intensive care unit; Preoperative area; Other

9. A8. Do you work shifts or on-call duties?

Response options: Yes; No

B. Institutional climate and awareness

Scale for items B1–B5: 1 = Strongly disagree; 2; 3; 4; 5 = Strongly agree

10. B1. My institution treats workplace sexual harassment as a serious problem.

11. B2. I believe that I would not face retaliation if I reported sexual harassment.

12. B3. I know how to report sexual harassment in my institution.

13. B4. Managers demonstrate appropriate role-model behavior regarding this issue.

14. B5. Support mechanisms for affected persons are sufficient in my institution.

C. Experiences during the previous 12 months

15. C1. During the previous 12 months, were you personally exposed to gender-based harassment or sexual harassment at work?

Response options: Yes; No; Prefer not to answer

16. C2. During the previous 12 months, did you witness sexual harassment directed at another person at work?

Response options: Yes; No; Unsure

17. C3. During the previous 12 months, how often did you experience or witness each of the following behaviors?

Behavior
Belittling or disrespectful words/behavior because of my sex or gender
Exclusion or being ignored because of my sex or gender
An uncomfortable comment about my appearance or private life

Behavior
A sexual joke or insinuation
Unwanted flirting, message, or offer
Unwanted physical contact

Response options for each row: Never; Once; 2–3 times; More than once per month; More than once per week

18. C4. Who was most often the source of these behaviors? Select all that apply.

Response options: Academic anesthesiologist; Anesthesiologist; Anesthesiology resident; Surgical physician; Operating-room nurse; Anesthesia technician/technologist; Administrative staff; Patient/patient relative; I did not experience such a situation

19. C5. In which setting did these behaviors occur most often?

Response options: Operating room; Intensive care unit; Ward/preoperative–postoperative area; Meeting or training setting; Digital setting (messaging, email, etc.); Academic setting/congress; I did not experience such a situation

If "I did not experience such a situation" was selected, the form routed the respondent to Question 29.

20. C6. What was the power relationship between you and the source/perpetrator?

Response options: The source was in a superior position; The source was at the same hierarchical level; The source was in a subordinate position; Patient/patient relative; I did not experience such a situation

If "I did not experience such a situation" was selected, the form routed the respondent to Question 29.

D. Effects

Scale for items D1–D6: 1 = Not at all; 2; 3; 4; 5 = Very much

21. D1. Increase in anxiety and stress

22. D2. Decrease in job satisfaction

23. D3. Symptoms of burnout

24. D4. Adverse effect on work performance

25. D5. Reduced trust in the institution or team

26. D6. Thoughts of leaving the job or changing institution

E. Reporting behavior

Routing note. Questions D1-D6 and E1-E2 were displayed only to respondents who did not select 'I did not experience such a situation' at the setting or power-relationship routing items. E1 referred globally to 'the situation that you experienced or witnessed' and did not identify a particular behavior item, event severity, or personal-experience versus witnessing status.

27. E1. Did you report the situation that you experienced or witnessed?

Response options: Yes; No; I considered reporting but did not

28. E2. For respondents who did not report: what was your primary reason for not reporting?

Response options: I thought nothing would change; Fear of retaliation; The source/perpetrator was in a powerful position; Lack of evidence; I did not know the reporting route

F. Prevention and recommendations

29. F1. Rate the effectiveness of each of the following practices in preventing sexual harassment.

**Prevention strategy**
Mandatory training programs
Anonymous reporting system
Protection against retaliation
Managerial sanctions
Psychological support services

Response options for each row: 1 = Ineffective; 2 = Slightly effective; 3 = Moderately effective; 4 = Effective; 5 = Very effective

30. F2. What is your most important recommendation for reducing this problem in your institution?

Open-text response. This field was technically mandatory. Respondents could enter a non-substantive response such as 'none' or punctuation if they did not wish to provide a recommendation.

Supplementary Table S1

Sex comparisons for perceived effectiveness of prevention strategies

Prevention strategy	Women median (IQR)	Men median (IQR)	Mann-Whitney U	p value	FDR q value
Mandatory training programs	2.0 (1.0-4.0)	3.0 (2.0-4.0)	3002.0	0.019	0.094
Anonymous reporting system	4.0 (2.5-4.0)	4.0 (3.0-4.0)	3778.0	0.991	0.991
Protection against retaliation	4.0 (3.0-5.0)	4.0 (3.0-5.0)	3754.0	0.952	0.991
Managerial sanctions	4.0 (4.0-5.0)	4.0 (3.0-5.0)	4209.0	0.173	0.431
Psychological support services	4.0 (3.0-5.0)	4.0 (2.0-5.0)	3964.0	0.563	0.938

Note: Scores ranged from 1 (ineffective) to 5 (very effective). p values are unadjusted two-sided Mann-Whitney U tests. FDR q values were calculated with the Benjamini-Hochberg procedure across the five prevention-strategy comparisons. Women n = 111; men n = 68. No comparison remained statistically significant after adjustment.
